# A Global Dataset of Potential Chloride Deposits on Mars as Identified by TGO CaSSIS

**DOI:** 10.1038/s41597-024-03685-3

**Published:** 2024-08-03

**Authors:** V. T. Bickel, N. Thomas, A. Pommerol, L. L. Tornabene, M. R. El-Maarry, V. G. Rangarajan

**Affiliations:** 1 Center for Space and Habitability, University of Bern, Bern, Switzerland; 2 Physikalisches Institut, University of Bern, Bern, Switzerland; 3 Institute for Earth and Space Exploration, University of Western Ontario, London, Canada; 4 The SETI Institute, Mountain View, CA USA; 5 Space and Planetary Science Center, Khalifa University, Abu Dhabi, UAE

**Keywords:** Mineralogy, Geomorphology, Computer science

## Abstract

Chloride deposits are markers for early Mars’ aqueous past, with important implications for our understanding of the martian climate and habitability. The Colour and Stereo Surface Imaging System (CaSSIS) onboard ESA’s Trace Gas Orbiter provides high-resolution color-infrared images, enabling a planet-wide search for (small) potentially chloride-bearing deposits. Here, we use a neural network to map potentially chloride-bearing deposits in CaSSIS images over a significant fraction of the planet. We identify 965 chloride deposit candidates with diameters ranging from <300 to >3000 m, including previously unknown deposits, 136 (~14%) of which are located in the highlands north of the equator, up to ~36°N. Northern chloride candidates tend to be smaller than in the south and are predominantly located in small-scale topographic depressions in low-albedo Noachian and Hesperian highland terranes. Our new dataset augments existing chloride deposit maps, informs current and future imaging campaigns, and enables future modelling work towards a better understanding of the distribution of near-surface water in Mars’ distant past.

## Background & Summary

Chloride-bearing deposits are mineralogical markers for Mars’ dynamic aqueous past, because they are highly soluble and, thus, record the last time a surface was covered by water. The deposits, and their depositional environments in particular, can provide optimal conditions for biological activity and preservation, which makes chloride-bearing terrain a prime target for astrobiological exploration^1,2^. First discovered by^2^, chlorides occur as decameter- to kilometer-sized deposits, often permeated with polygonal fractures and/or ridges^3–5^ that tend to be located in local topographic depressions^1,6^ and sometimes are associated in channel-like features and valley networks^4,7^.

So far, chlorides are predominantly identified in the southern highlands of Mars^2,4–6,8^. This has strongly influenced hypotheses about their formation and evolution. For example^6^, suggest that the distribution of chlorides in the south loosely follows present-day Mars’ zone of maximum expected precipitation^9^, which in turn points to a predominantly Hesperian formation age for chlorides. Independent of their age, most authors agree that chlorides form as a result from evaporation/precipitation of ponded water from near-surface run-off and/or groundwater up-welling^3,4,7,10^. Other, alternative formation scenarios include diagenetic and/or hydrothermal brines, efflorescent crust, and deep lakes^11,12^.

**Fig. 1:**
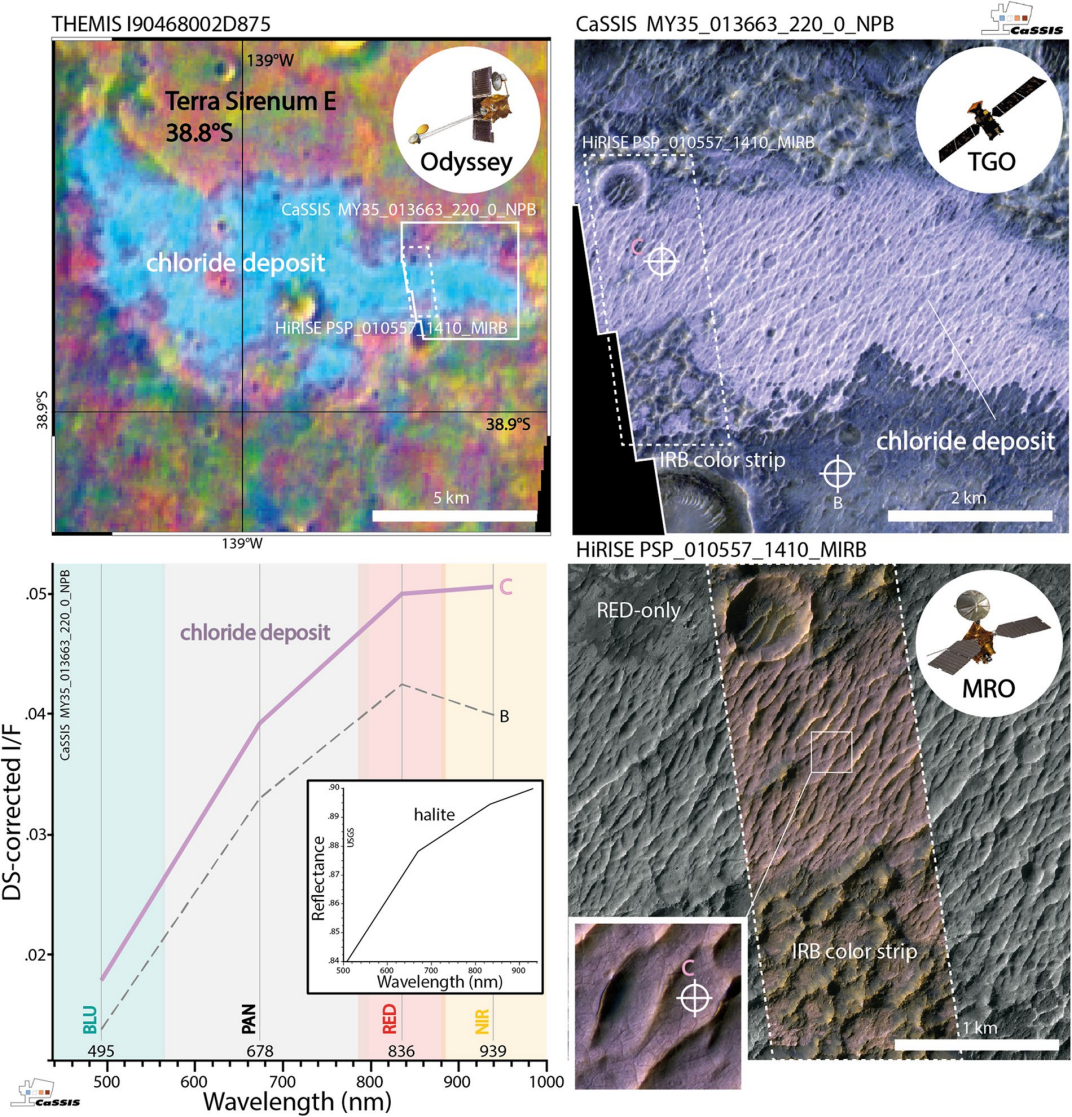
Martian chloride deposits as imaged by different orbital sensors. Top left; Mars Odyssey THEMIS 875 DCS (decorrelation stretched) false-color image of the original chloride deposit (light blue) characterized by^2^ in East Terra Sirenum, located close to the CRISM Terra Sirenum chloride type locality (31.6°S, 206.4°E) characterized by^36^; TGO (Trace Gas Orbiter) CaSSIS and MRO (Mars Reconnaissance Orbiter) HiRISE image footprints outlined in white. Top right, bottom right; CaSSIS NPB (near-infrared, panchromatic, blue-green) and HiRISE MIRB (infrared, red, blue-green) acquisitions of a section of the same deposit (note the limited width of the HiRISE IRB color strip). Bottom left; DS-corrected (Dark-object Subtraction^37,38^ spectra of the chloride (C) and one background location (B), see markers in the CaSSIS and HiRISE images. Note the distinct similarities between the DS-corrected CaSSIS chloride spectrum and the USGS halite reference spectrum, particularly the positive RED-NIR slope (^39^, resampled to CaSSIS wavelengths). This example showcases that CaSSIS color-infrared data can be used to identify potentially chloride-bearing terrain, similar to HiRISE MIRB, acknowledging the remaining ambiguity. Image credit: ESA/TGO/CaSSIS CC-BY-SA 3.0 IGO, NASA/JPL/University of Arizona/Arizona State University.

Chlorides are light-toned and typically have a characteristic pink to violet hue in color-infrared images (false color composites using a combination of near-infrared, ~900 nm, red, ~670 nm, and blue channels, ~500 nm), which makes them readily recognizable from orbit (Fig. 1). In the absence of color, chlorides are easily confused with other light-toned materials such as clays^6,8^, meaning that the unambiguous detection and classification of chlorides requires (1) multi-spectral (color-infrared) information. At the same time, (2) high to intermediate spatial resolution (<~10 meters per pixel) is required to recognize smaller deposits^10^. Any global-scale study further requires (3) extensive image coverage of the martian surface. However, as of today, there exists no image dataset that meets all three criteria.

Past global-scale mapping efforts have relied on either THEMIS (Thermal Emission Imaging System), CRISM (Compact Reconnaissance Imaging Spectrometer for Mars), OMEGA (Observatoire pour la Minéralogie, l’Eau, les Glaces et l’Activité), and/or HiRISE (High Resolution Imaging Science Experiment) data^2,4,6^. THEMIS and OMEGA provide global coverage but lack spatial resolution (THEMIS > ~100 m/pixel; OMEGA > ~300 to ~5,000 m/pixel), and CRISM and HiRISE provide intermediately high to high spatial resolution (CRISM > ~18 to ~200 m/pixel; HiRISE ~0.3 m/pixel) but lack spatial coverage (only <3% and ~0.7% of the surface covered with multispectral data, respectively – note that HiRISE provides only one ~1.2 km-wide color strip in the center of the image) (e.g.^13^).

**Fig. 2:**
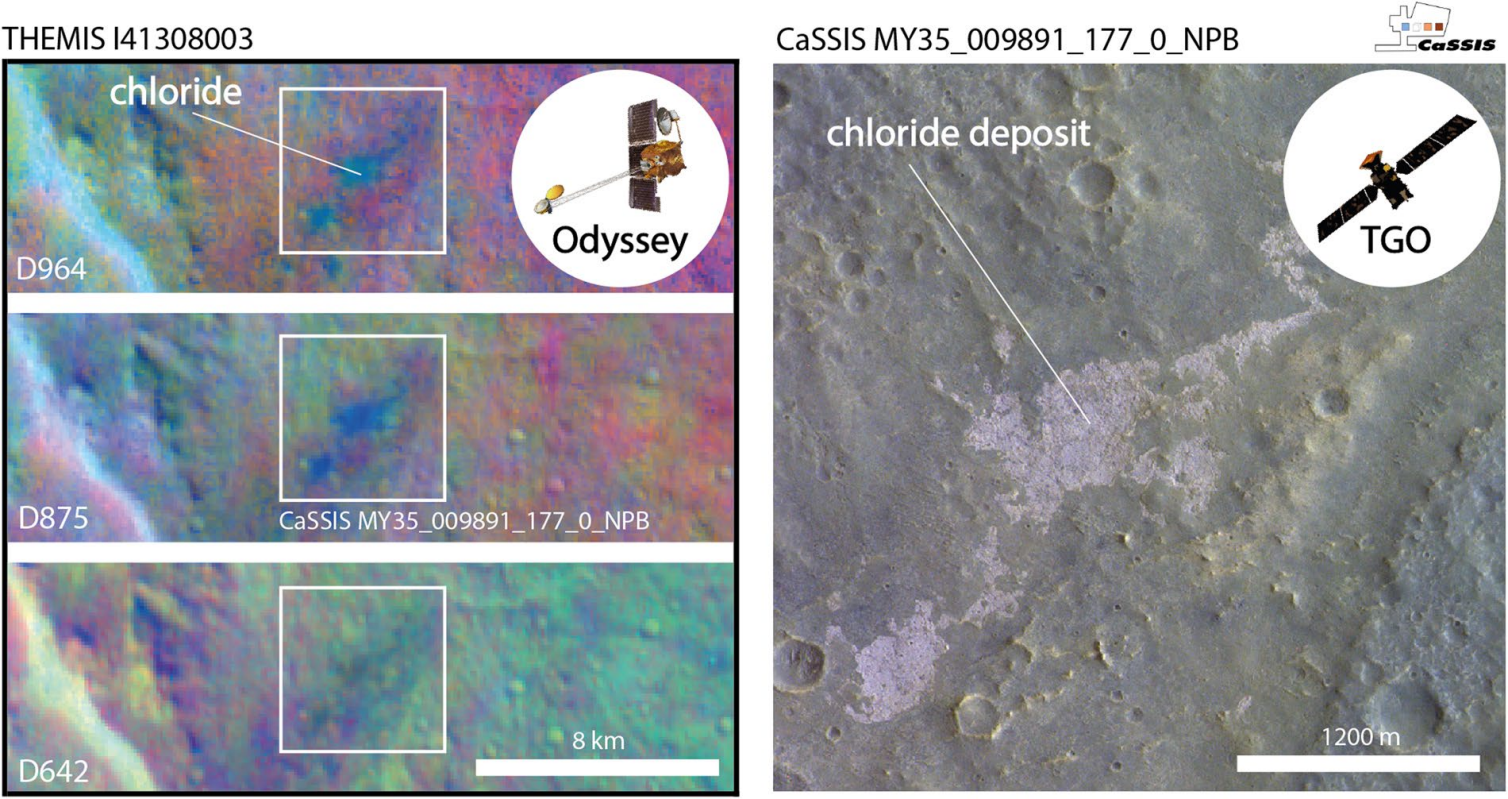
Example of a chloride-bearing deposit missed by earlier surveys but identified by CaSSIS. Left; THEMIS 964, 875, and 642 DCS (decorrelation stretched) false-color images of a chloride deposit not reported in the^4^ database; CaSSIS image footprint outlined in white. In THEMIS DCS products chloride-bearing terrain appears teal, blue, and yellow/orange in 964, 875, and 642, respectively. Right; CaSSIS NPB (near-infrared, panchromatic, blue-green) acquisition of the same deposit. This example showcases that CaSSIS color data can be used to detect potentially chloride-bearing terrain, acknowledging the remaining ambiguity (in this specific case the THEMIS DCS data strongly suggests the presence of chloride). Image credit: ESA/TGO/CaSSIS CC-BY-SA 3.0 IGO, NASA/JPL/Arizona State University.

Besides the limitations of the data, previous studies relied on manual mapping efforts (e.g.^2,4,6^), which might suffer from a variety of observational biases related to, for example, operator awareness fatigue, operator-to-operator expertise differences, and observer expectancy effects, potentially resulting in incomplete and/or inconsistent mapping results (Fig. 2). Notably^14^, built a machine learning-based, spectral variability-focused classification routine for CRISM data. However, this routine suffers from the same, instrument-related limitations mentioned above (lack of spatial resolution and coverage), while not being able to draw from any morphological information that is also encoded in the CRISM image data. As a consequence of the limitations of the data and mapping methods, past efforts might have missed – small as well as relatively large – chloride-bearing deposits, with potentially important implications for the derived conclusions regarding Mars’ past climate and evolution.

In this work, we address some of the limitations related to the data and the mapping methodology by (1) utilizing the new color-infrared (BLU – green/blue, PAN - panchromatic, RED – far-red, NIR – near-infrared, see Fig. 1) and high-resolution (~4 m/pixel) image data acquired by ESA Trace Gas Orbiter’s (TGO) CaSSIS (Colour and Stereo Surface Imaging System, https://observations.cassis.unibe.ch/)^15^ instrument and by (2) employing a reliable and rapid few-shot learning-driven detection and mapping workflow that can effectively draw from spectral as well as morphological information encoded in CaSSIS images. To date, CaSSIS data has not been used to systematically map chloride-bearing deposits on Mars. It is important to note that CaSSIS is not able to meet all of the three main mapping requirements either: the data features high spatial resolution (~4 m/pixel), but has only covered ~7.1% of the surface to date; in addition, its 4 spectral bands (BLU, PAN, RED, NIR) allow for a detection of potentially chloride-bearing terrain, but do not provide a sufficient amount of spectral resolution and range to enable an unambiguous identification of chloride. In turn, THEMIS and OMEGA are spatially not highly enough resolved to enable a characterization of the majority of the small, potentially chloride-bearing deposits identified in CaSSIS data, rendering a thorough verification of the CaSSIS detections impossible. Throughout this manuscript we provide qualitative evidence for a good agreement between large CaSSIS-detected and THEMIS-detected chloride deposits (Figs. 1,2), but – due to the remaining ambiguity –we refer to all CaSSIS detections as “chloride deposit candidates” or “potential chloride-bearing deposits”, pending future verification.

**Fig. 3:**
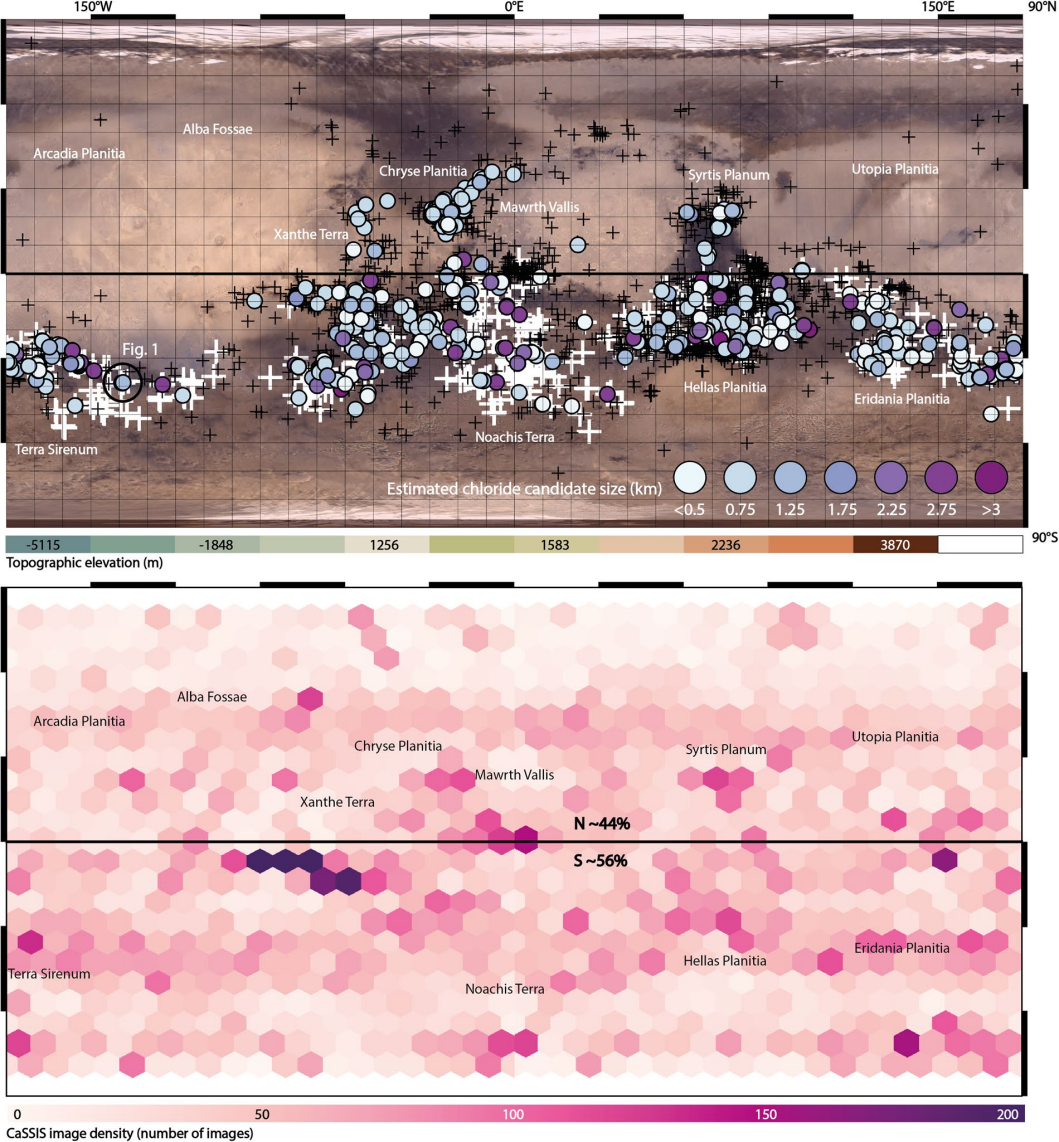
The distribution of potential chloride-bearing deposits on Mars as identified by CaSSIS. Top: Global distribution of all CaSSIS-derived chloride candidates (shades of violet); color indicates the estimated size of the respective candidate; white/black crosses mark the locations of THEMIS-/OMEGA-derived chloride deposits/hydrous mineral detections^4,35^; Viking Merged Color Mosaic in the background. Note mark for the location of Fig. 1 (black circle). Bottom: coverage heatmap (hexmap) of CaSSIS image density including orbit number 27,816, 02/17/2024.

Our dataset includes a total of 965 potentially chloride-bearing deposits on the martian surface, identified in a total of 487 CaSSIS images (Fig. 3). Notably, about 14% (n = 136) of the potential chloride deposits are located in the highlands north of the equator (up to ~35°N), representing the first-ever detections in the northern hemisphere, if verified by future missions and efforts. We note that northern chloride candidates appear to be more obscured and spatially discontinuous than in the south, potentially indicating more degradation (weathering and erosion) in these areas. The identified chloride deposit candidate feature sizes range from <300 to >3000 meters across, with a mean of 1043 m. About 60% of all candidates are located on Noachian terranes, while about 38% are located on Hesperian terranes; only 2% are located on Amazonian terranes (Fig. 3). We note that the candidates on Amazonian terranes appear to be larger than in other terranes, on average, but acknowledge the small sample size. The majority of the identified chloride candidates are located within sinuous channels, valleys, and along the edges of local topographic lows, but we also identify a number of chloride candidates in local topographic highs - on the slopes and on surfaces above valleys, as well as on massifs and mesas -, suggesting those deposits formed before weathering and erosion formed the valleys, as found by earlier surveys, too (e.g.^6^). We note that our dataset includes potential chloride deposits missed by earlier efforts, such as in Mawrth Vallis, Vichada Valles, Nirgal Vallis, and Syrtis Major (Figs. 2, 3).

Our dataset provides new, global-scale insights into the distribution and properties of chlorides on Mars, augments existing chloride deposit datasets, and enables future modelling efforts towards a better understanding of the presence of near-surface water in Mars’ distant past and – thus – Mars’ past habitability. In addition, this dataset can be used to directly inform future high-resolution and multi-/hyper-spectral imaging campaigns as well as concept studies for future landed missions.

## Methods

### Few shot learning-driven mapping of potentially chloride-bearing deposits

**Fig. 4:**
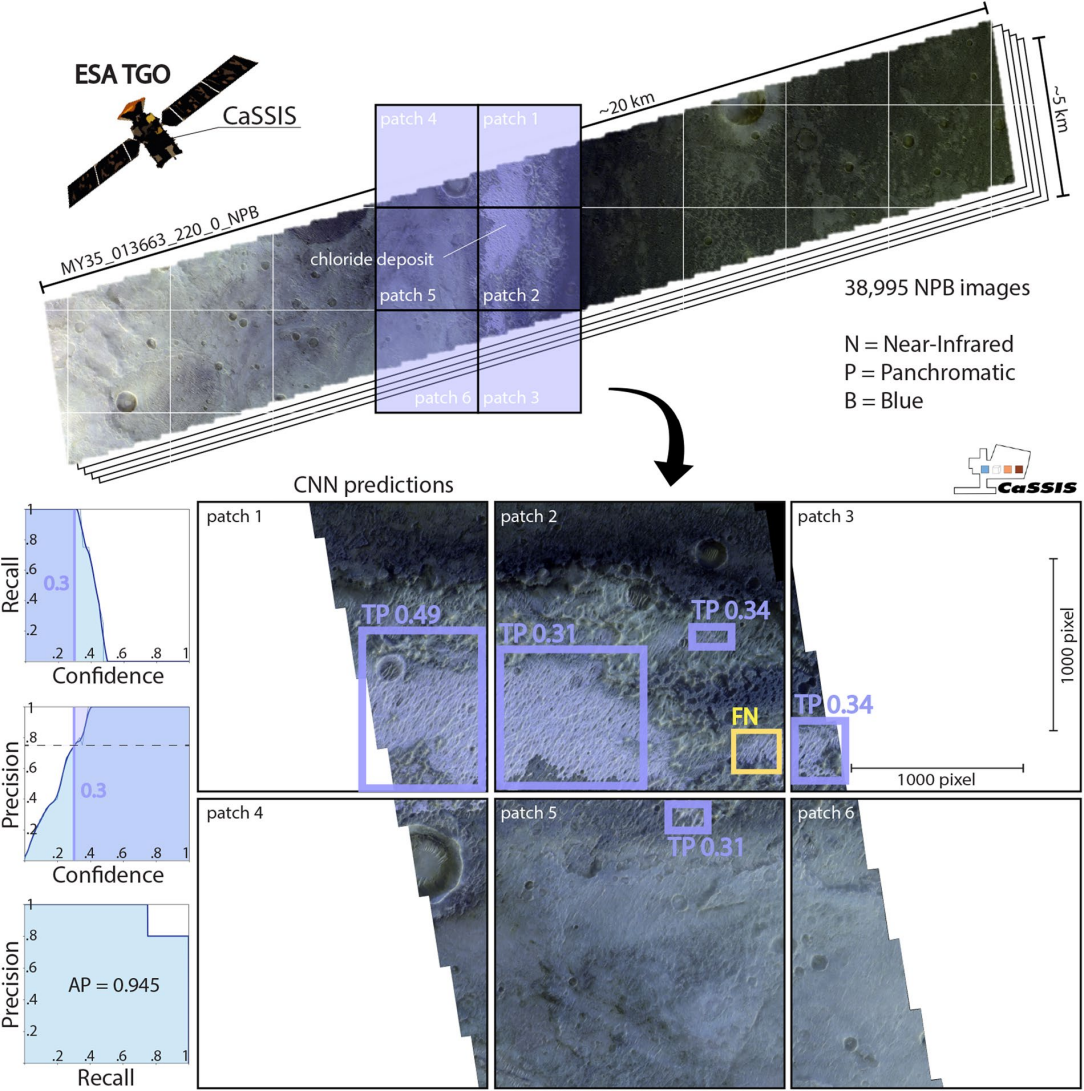
Schematic of CaSSIS chloride deposit mapping workflow. CaSSIS NPB images (n = 38,995) are patched and input into the CNN; the CNN predicts chloride locations (boxes) and scores (indicated next to boxes), including true positives (TPs, violet), false negatives (FNs, yellow), and false positives (FPs, not present here); the detector achieves an AP of 94.5% in the testset (bottom left); deployment CT cut-off at 0.3 is indicated by a vertical violet line on the left. Image credit: ESA/TGO/CaSSIS CC-BY-SA 3.0 IGO.

**Fig. 5:**
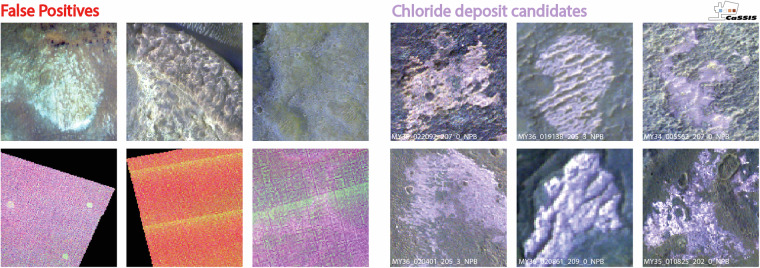
Examples of false and (presumably) correct CaSSIS chloride detections. Left: examples of false detections, such as clay deposits and imaging artefacts; right: examples of correct chloride deposit candidate detections. Image credit: ESA/TGO/CaSSIS CC-BY-SA 3.0 IGO.

We train and test a convolutional neural network (CNN) using a total of twenty-nine calibrated and map-projected CaSSIS NPB (NIR, PAN, BLU) images^16,17^ (Table 1), consisting of all appropriate images of chloride (candidates) known to the CaSSIS science team as of early 2023 (few-shot learning). The combination of its distinct morphological characteristics and spectral properties (i.e., pink to violet hue in CaSSIS color-infrared NPB products, Figs. 1, 2) permits the detection of potentially chloride bearing-terrains. Notably, initial experiments with simple pixel-based detectors failed due to the non-uniqueness of chloride-colored pixels (pink to violet hue). Ultimately, the complexity of the CaSSIS dataset, as caused by the heterogeneous martian surface and atmosphere, directly led us to using a CNN-based approach. The qualitative agreement of CaSSIS- and THEMIS-derived (large) chloride detections are showcased in Figs. 1 and 2. We use the openly available YOLOv5 object detection architecture and YOLOv5x model, distributed by ultralytics on GitHub (https://github.com/ultralytics/yolov5). YOLOv5 is a cutting-edge and well-established object detection framework that outperforms (regarding speed and performance) numerous other networks on benchmarks such as COCO (Common Objects in Context). YOLOv5 has successfully been used across a number of scientific fields, including medical imaging^18^, food science^19^, robotics^20^, and planetary science^21^. Following routines established in earlier work^22–25^, we manually label all chlorides that are visible in the available CaSSIS images, resulting in a total of 61 individual labels distributed over a total of 58 patches (a patch is a CaSSIS image crop-out with a size of about ~1000 by ~1000 pixels). Here, a label represents a rectangular bounding box that encompasses a chloride deposit, drawn by a human operator using the supervise.ly platform (Fig. 4). The term ‘encompass’ refers to the fact that the bounding box contains the majority of the surface-exposed area of a given chloride bearing deposit; we note that the shape of chloride-bearing deposits can be highly irregular and may not always be properly characterized by a rectangular bounding box (see Figs. 4 and 5).

**Table 1:** List of all CaSSIS images used for chloride detector training and evaluation.

MY36_021497_337_3_NPB	MY35_008275_165_0_NPB	MY34_003635_325_1_NPB
MY34_003867_340_0_NPB	MY34_004065_215_1_NPB	MY34_004450_222_1_NPB
MY34_005755_222_0_NPB	MY35_011355_319_0_NPB	MY35_011567_318_0_NPB
MY35_012172_214_0_NPB	MY35_012286_213_0_NPB	MY35_012818_328_0_NPB
MY35_012905_329_0_NPB	MY35_013129_349_0_NPB	MY35_013222_331_0_NPB
MY35_013651_213_0_NPB	MY35_013664_214_0_NPB	MY35_013751_213_0_NPB
MY35_013920_327_0_NPB	MY35_014111_339_0_NPB	MY36_015156_216_0_NPB
MY36_016614_199_0_NPB	MY36_019273_213_1_NPB	MY36_019307_221_0_NPB
MY36_019444_213_0_NPB	MY36_019833_346_0_NPB	MY36_019975_325_1_NPB
MY36_021144_325_0_NPB	MY36_021375_328_3_NPB	

CaSSIS NPB data is available here: https://observations.cassis.unibe.ch/. MY = Mars Year.

We include a total of 56 negative image patches (without any chlorides) to enable the CNN to learn differences between (pink/violet) chlorides and atmospheric/geologic and imaging-related artefacts, such as color fringes caused by moving clouds and spatial color channel misalignments. We use label and data augmentation to prevent overfitting and compensate for the small number of training labels, specifically using label rotation, left-right/up-down flipping, shearing, up- and down-scaling ($\pm$ 10%), as well as contrast and brightness modifications. We note that up- and down-sampling augmentation operations are not able to bridge the gap in spatial resolution between the different available, multi-spectral remote sensing datasets as introduced above. However, the augmentation of the spatial resolution ($\pm$ ~0.4 m/pixel) helps to make the detector more robust against small variations in the spatial resolution of the CaSSIS image data (nominally ~4 m/pixel).

From the overall labeled set, we randomly draw a small testset of eight patches and 4 labels, representing about ~7% of the overall dataset. We deliberately minimize the size of the testset to maximize the size of the data that were available for training, given the scarcity of known, verified, CaSSIS-observed chlorides. We note that the testset size used for detector evaluation in planetary science applications is usually ~5 to ~10% of the overall available data (e.g.^21,26,27^). The testset is omitted during the training of the CNN. The trained CNN achieves a very high average precision (AP) of 94.5%, achieving total recall (100%) at a confidence of about 35% and total precision (100%) at a confidence of about ~41% (Fig. 4), using an IoU (Intersection of Union) of 0.5. Here, recall describes the number of successfully detected features and precision describes the percentage of correct detections. The term confidence quantifies a detection’s posterior probability as assigned by the CNN (ranging from 0, low, to 100%, high). The evaluation suggests that we can expect the detector to identify the vast majority of all CaSSIS-observed chloride-bearing deposits while producing an acceptable number of false positives at a confidence score between ~0.3 to ~0.4. Based on past experience, we caution that testset evaluation usually overestimates the true performance of the detector in the actual, full dataset^27,28^.

The evaluated detector is deployed in an existing processing pipeline^29^ that streams and processes 38,995 CaSSIS NPB images (representing ~89% of the total dataset, up to orbit number 27,816, 02/17/2024) at a rate of ~20 images per minute using one NVIDIA GeForce RTX 3090 graphical processing unit (GPU). Prior to CNN-processing, each CaSSIS image is patched into 1000 × 1000 pixel tiles, to avoid GPU memory-overflow issues. We deploy the detector using a confidence threshold (CT) of 30%, effectively maximizing the precision (~78% in the testset) while maintaining total recall (100% in the testset).

## Data Records

The catalog of all CaSSIS-derived, potentially chloride-bearing deposits is openly available here: 10.48620/352^30^ (‘*Bickel_et_al_2024_chloride_CT03’*). Future releases of the CaSSIS chloride-bearing terrain database will be made available under the same DOI but with unique version identifiers (‘*Bickel_et_al_2024_chloride_CT03_vn.n’*).

The outputs of the processing pipeline include full-resolution crops (thumbnails) of all detected candidate chlorides as well as a rich set of metadata, including geographic information and CNN-derived information.

### Metadata

All CaSSIS-identified chloride deposit candidates are reported in one single.csv file, which includes 23 separate metadata columns, providing geographic and CNN-derived information:

- **cassis_run_ID:** the internal detection ID
- **cassis_ID:** the ID of the CaSSIS image (MY….NPB_browse.png)
- **cassis_ID_short:** the short ID of the CaSSIS image (MY….)
- **date_time:** the datetime string of the CaSSIS image
- **center_LON_180d:** the longitude (180° domain) of the center of the detection in degree
- **center_LAT_pc:** the planetocentric latitude of the center of the detection in degree
- **chloride_diameter_meter:** estimated chloride deposit size in meter (using the diameter of the CNN-predicted bounding box; expected to overestimate deposit size, see e.g. Figure 5)
- **upper_left_x_img:** the x image coordinate of the NW corner of the detection in pixel
- **upper_left_y_img:** the y image coordinate of the NW corner of the detection in pixel
- **lower_right_x_img:** the x image coordinate of the SE corner of the detection in pixel
- **lower_right_y_img:** the y image coordinate of the SE corner of the detection in pixel
- **center_y:** the y image coordinate of the center of the detection in pixel
- **center_x:** the x image coordinate of the center of the detection in pixel
- **confidence:** the confidence score as achieved by the CNN
- **cassis_resolution:** the spatial resolution of the CaSSIS image (m/pixel)
- **emission:** the emission angle of the CaSSIS image in degree (0° = nadir-looking; 90° = horizontally-looking)
- **incidence:** the solar incidence angle of the CaSSIS image in degree (0° = vertical illumination; 90° = horizontal illumination)
- **phase:** the solar phase angle of the CaSSIS image in degree (0° = illumination aligned with viewing direction; 180° = illumination opposite to viewing direction)
- **sub_solar_longitude**: sub-solar longitude (i.e., season) of CaSSIS image in degree
- **local_time:** the local Mars time in 24:00 h (12:00 = noon)
- **num_filters:** the number of filters available for the CaSSIS image (3 or 4)
- **type:** the imaging mode used for the CaSSIS image (stereo pair observations are available for some locations)
- **thumbnail_ID:** the ID tag of the detection thumbnail

We provide an associated .shp file with the identical information, as an alternative way of loading and using the dataset.

### Thumbnails

Each identified chloride candidate features a full-resolution, KB-sized NPB.tif thumbnail, as cropped from its respective parent CaSSIS NPB image (see Fig. 5). The name tag of each thumbnail is included in the metadata file, as described above.

## Technical Validation

The CNN-derived dataset is subject to a two-pronged review and validation approach, including 1) expert (manual) review and 2) a spatial and geostatistical comparison with chloride deposit datasets derived by earlier surveys using different image data.

### Expert review of all identified chloride candidates

According to the testset performance (Fig. 4), the detector should reach total recall at a very high precision. We conduct an expert-driven, case-by-case, manual review of all CNN-derived candidates to verify the performance of the detector as based on the testing results and remove all obvious false detections, i.e., candidates that do not closely resemble pink- to violet-hued and textured chloride-bearing deposits (Fig. 5). During review, an expert visually examines every single detected candidate thumbnail as well as the overall geologic context, if required. During this process, we remove ~50% of all detected chloride candidates (false positives), indicating that the real world performance of the detector is significantly lower than its testset performance.

The majority of the removed false positives are related to images with artefacts and particularly poor signal-to-noise ratios (e.g., color-channel misalignments, early morning images, Fig. 5), likely because they are underrepresented in the training and testing datasets (a complete list of low quality images was not available as of training/testing). The discrepancy between test-time and inference (full dataset deployment) performance is common (e.g.^27,28^) and can usually be attributed to the overall lack of training and testing data and/or a general lack of “detector dataset awareness”, i.e., a detector’s incomplete understanding of the full variability of the target’s geomorphic and spectral appearance, potential look-alikes (e.g., potentially pinkish/reddish/white-bluish mineral deposits), and the frequency and nature of imaging artefacts.

### Spatial and geostatistical comparison with other chloride deposit datasets

We compare the expert-reviewed chloride deposit candidates with MOLA (Mars Orbiter Laser Altimeter) topographic elevation data^31^, TES (Thermal Emission Spectrometer) surface albedo^32^, the latest revision of the martian geologic map^33^, as well as with the global CTX mosaic as provided by^34^. In addition, we compare our results with THEMIS-derived chloride maps^4,6^ and CRISM- and OMEGA-derived hydrous mineral detections^35^, which are able to thoroughly identify chloride, but only at low spatial resolution, i.e., are likely to lack small chloride deposits. Notably, OMEGA hydrous mineral detections not only include chloride, but predominantly other minerals like phyllosilicates, smectites, and serpentines^35^.

**Fig. 6:**
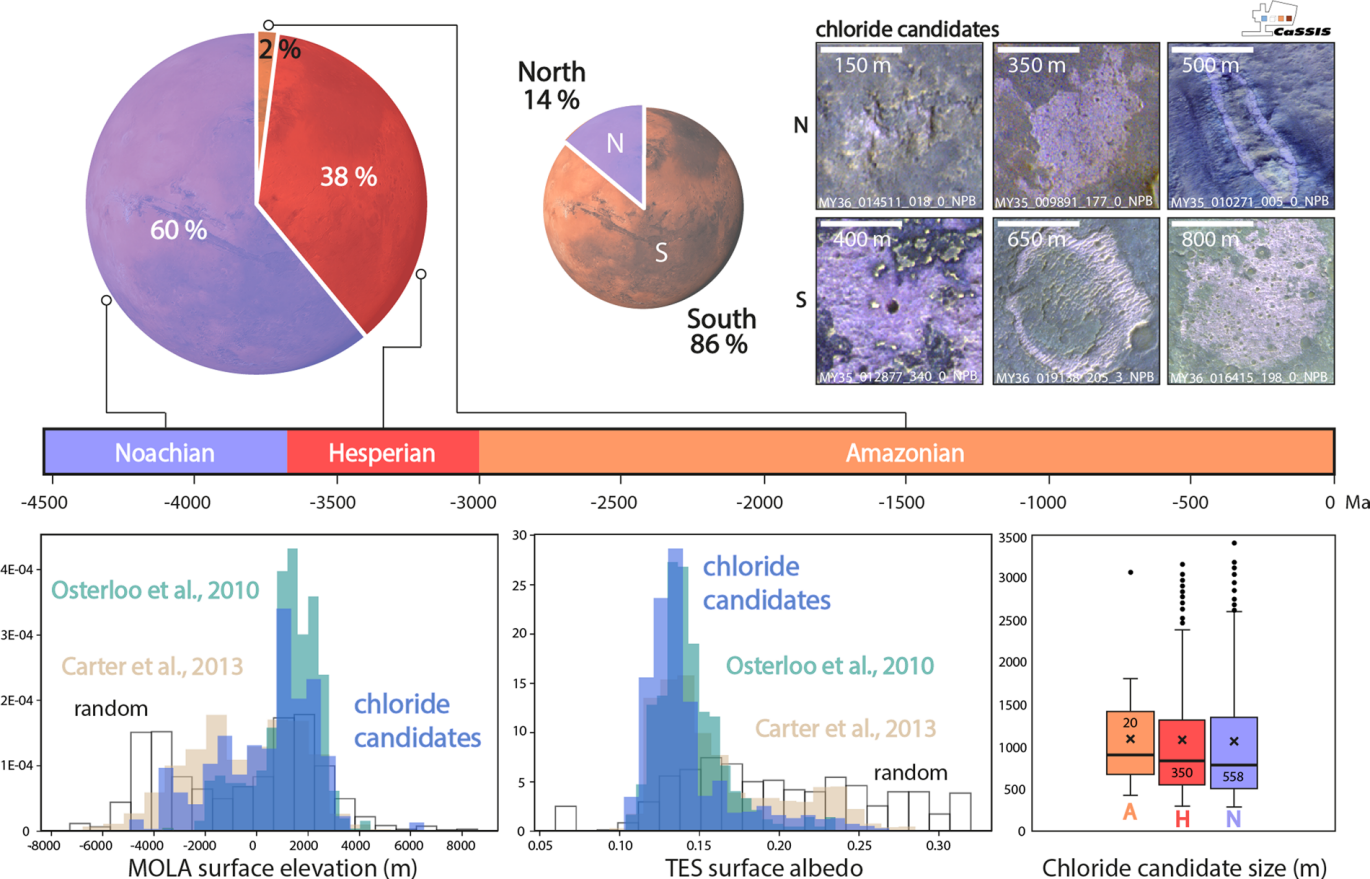
Spatial and geostatistical validation of the CaSSIS chloride candidate dataset. Top left; chloride distribution as a function of host terrane age (terrane age taken from^33^); top center; distribution over the northern and southern hemispheres; top right; examples of northern (top) and southern (bottom) chloride candidates as observed by CaSSIS; center; distribution of MOLA topographic elevation and TES surface albedo for (**a**) chlorides (violet), (**b**) THEMIS chloride detections (tale^4^), (**c**) OMEGA hydrous mineral detections (tan^35^), and **d**) 1200 random points (black); center right; box plots for chloride size and host terrane age. Note that the pie charts’ Mars background serves visualization purposes only. Image credit: ESA/TGO/CaSSIS CC-BY-SA 3.0 IGO.

**Fig. 7:**
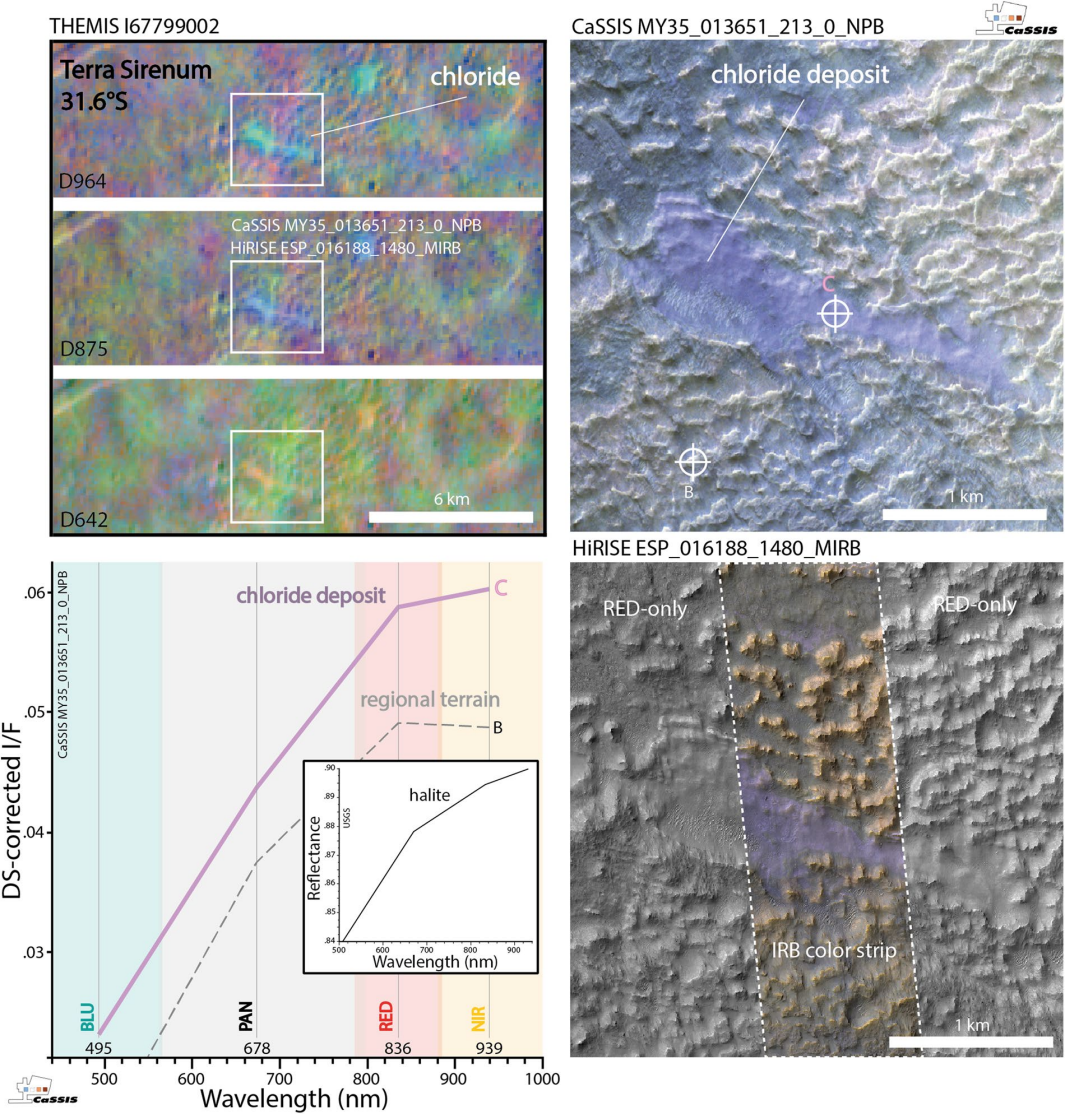
CaSSIS detection of the CRISM chloride type locality. Top left; THEMIS 964, 875, and 642 DCS (decorrelation stretched) images of the CRISM chloride type locality in Terra Sirenum (31.6°S, 206.4°E) characterized by^36^; CaSSIS and HiRISE image footprints outlined in white. In THEMIS DCS products chloride-bearing terrain appears teal, blue, and yellow/orange in 964, 875, and 642, respectively. Top right, bottom right; CaSSIS NPB (near-infrared, panchromatic, blue-green) and HiRISE MIRB (infrared, red, blue-green) acquisitions of a section of the same deposit (note the limited width of the HiRISE IRB color strip). Bottom left; DS-corrected (Dark-object Subtraction^37,38^) spectra of the chloride (C) and one background location (B), see markers in the CaSSIS and HiRISE images. Note the distinct similarities between the DS-corrected CaSSIS chloride spectrum and the USGS halite reference spectrum, particularly the positive RED-NIR slope (^39^, resampled to CaSSIS wavelengths). This example showcases that CaSSIS color-infrared data can be used to identify chloride-bearing terrain, similar to HiRISE MIRB, as further showcased by Figs. 1 and 2. Image credit: ESA/TGO/CaSSIS CC-BY-SA 3.0 IGO, NASA/JPL/University of Arizona/Arizona State University.

**Fig. 8:**
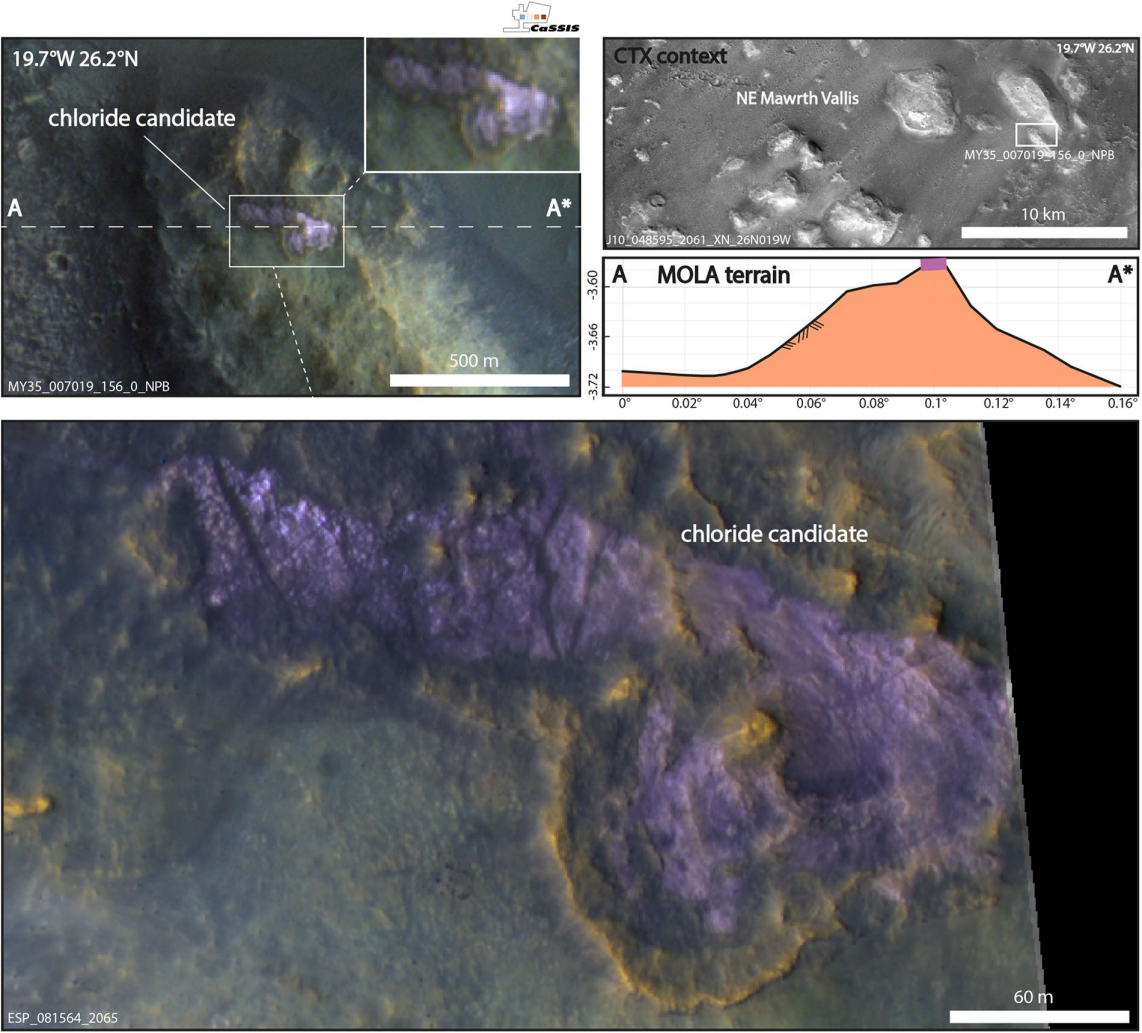
CaSSIS detection on a mesa in Mawrth Vallis. Top left; CaSSIS-detected chloride candidate located on a mesa. Top right; geomorphic context and topographic profile as provided by CTX and MOLA; cross-section indicated by white dashed line (A-A*) on the CaSSIS image. Bottom; the CaSSIS-detected chloride candidate as re-imaged by HiRISE. Image credit: ESA/TGO/CaSSIS CC-BY-SA 3.0 IGO, NASA/JPL/University of Arizona/Arizona State University.

The spatial distribution of CaSSIS-derived chloride candidates agrees well with previous surveys^2,4,6^ as well as with CRISM & OMEGA hydrous mineral detections^35^ (Fig. 3). We note that the candidates seem to cluster on surfaces with very similar albedo as the THEMIS-derived chloride detections mapped by^4^ (Fig. 6). Similarly, the CaSSIS candidates occur within the same elevation range as the THEMIS detections, while expanding to slightly lower elevations (~2500 to ~4000 m), due to the small portion of northern candidates contained in the CaSSIS data. The OMEGA-derived hydrous mineral detections^35^ agree with the CaSSIS data as well, with slight differences at very high albedo and low elevations, likely explained by non-chloride related OMEGA detections in the northern hemisphere (e.g. in Arabia Terra). These results provide geostatistical evidence that the majority of the CNN-derived chloride candidates mapped in CaSSIS images in fact represent chloride-bearing terrain. Figure 7 showcases another direct comparison of the CRISM chloride type locality located in Terra Sirenum (31.6°S, 206.4°E^36^) that was independently detected in CaSSIS. We note that the CaSSIS-derived dataset does not include all of the locations hosting chloride-bearing terrain identified by earlier efforts, such as located in Noachis Terra, Icaria Fossae, and Tader Valles; the vast majority of these “misses” can be explained by a) a lack of CaSSIS images over these locations and b) poor CaSSIS data quality as caused by, e.g., atmospheric processes, illumination issues, or data acquisition artefacts (see Fig. 5). We note that – vice versa – the CaSSIS-derived dataset includes (previously unknown/un-reported) potentially chloride-bearing terrain not included in earlier datasets, such as in Mawrth Vallis, Vichada Valles, Nirgal Vallis, and Syrtis Major. The vast majority of the newly identified, potentially chloride-bearing deposits in those regions are small (<~1 km, Fig. 3), which might explain why they were missed by earlier surveys. One of the small, previously un-reported candidates in Mawrth Vallis is showcased in Fig. 8.

## Usage Notes

The catalog of identified chloride deposit candidates can be displayed by every regular Geographic Information system (GIS) software, such as Quantum GIS (QGIS) and ArcGIS, either by importing the .csv file as a delimited text file or by dragging and dropping the associated .shp file into the GIS’ GUI.

The thumbnail records can be viewed with every regular image viewer. We recommend using the full CaSSIS image whenever studying a specific chloride deposit candidate, to maximize the geologic context available for interpretation. CaSSIS image data is available here: https://observations.cassis.unibe.ch/.

## Data Availability

This work is exclusively based on open software and data. The used object detection architecture (based on PyTorch) is openly available here (Python): https://github.com/ultralytics/yolov5. CaSSIS image data are openly available here (use the search bar to find specific image IDs or zoom and pan on the map): https://observations.cassis.unibe.ch/. HiRISE data are openly available here (use the search bar to find specific image IDs or use the “Map” function): https://www.uahirise.org/. CRISM data are openly available here (zoom and pan across the map to find images in specific regions, query using the “information” tool): http://crism-map.jhuapl.edu/. The used USGS map products (MOLA topography, TES albedo, geologic map) are openly available here, respectively: (1) https://astrogeology.usgs.gov/search/details/Mars/GlobalSurveyor/MOLA/Mars_MGS_MOLA_DEM_mosaic_global_463m/cub, (2) https://astrogeology.usgs.gov/search/map/Mars/GlobalSurveyor/TES/Mars_MGS_TES_Albedo_mosaic_global_7410m, (3) https://astrogeology.usgs.gov/search/map/Mars/Geology/Mars15MGeologicGISRenovation?p=2&pb=1#downloads. The used CRISM/OMEGA products are openly available here: https://www.cosmos.esa.int/web/psa/mars-maps.
